# Gankyrin inhibits ferroptosis through the p53/SLC7A11/GPX4 axis in triple-negative breast cancer cells

**DOI:** 10.1038/s41598-023-49136-8

**Published:** 2023-12-08

**Authors:** Ming Lei, Yun-Long Zhang, Feng-Ying Huang, Heng-Yu Chen, Ming-Hui Chen, Ri-Hong Wu, Shu-Zhen Dai, Gui-Sheng He, Guang-Hong Tan, Wu-Ping Zheng

**Affiliations:** 1 Department of Breast and Thyroid Surgery, The Second Affiliated Hospital, Hainan Medical University, Haikou, 570311 China; 2 Key Laboratory of Tropical Translational Medicine of Ministry of Education & School of Tropical Medicine, Hainan Medical University, Haikou, 571199 China

**Keywords:** Cancer, Cell biology, Computational biology and bioinformatics, Molecular biology, Biomarkers, Diseases, Medical research, Oncology

## Abstract

Gankyrin is found in high levels in triple-negative breast cancer (TNBC) and has been established to form a complex with the E3 ubiquitin ligase MDM2 and p53, resulting in the degradation of p53 in hepatocarcinoma cells. Therefore, this study sought to determine whether gankyrin could inhibit ferroptosis through this mechanism in TNBC cells. The expression of gankyrin was investigated in relation to the prognosis of TNBC using bioinformatics. Co-immunoprecipitation and GST pull-down assays were then conducted to determine the presence of a gankyrin and MDM2 complex. RT-qPCR and immunoblotting were used to examine molecules related to ferroptosis, such as gankyrin, p53, MDM2, SLC7A11, and GPX4. Additionally, cell death was evaluated using flow cytometry detection of 7-AAD and a lactate dehydrogenase release assay, as well as lipid peroxide C11-BODIPY. Results showed that the expression of gankyrin is significantly higher in TNBC tissues and cell lines, and is associated with a poor prognosis for patients. Subsequent studies revealed that inhibiting gankyrin activity triggered ferroptosis in TNBC cells. Additionally, silencing gankyrin caused an increase in the expression of the p53 protein, without altering its mRNA expression. Co-immunoprecipitation and GST pull-down experiments indicated that gankyrin and MDM2 form a complex. In mouse embryonic fibroblasts lacking both MDM2 and p53, this gankyrin/MDM2 complex was observed to ubiquitinate p53, thus raising the expression of molecules inhibited by ferroptosis, such as SLC7A11 and GPX4. Furthermore, silencing gankyrin in TNBC cells disrupted the formation of the gankyrin/MDM2 complex, hindered the degradation of p53, increased SLC7A11 expression, impeded cysteine uptake, and decreased GPX4 production. Our findings suggest that TNBC cells are able to prevent cell ferroptosis through the gankyrin/p53/SLC7A11/GPX4 signaling pathway, indicating that gankyrin may be a useful biomarker for predicting TNBC prognosis or a potential therapeutic target.

## Introduction

Triple-negative breast cancer (TNBC) is a particularly aggressive form of breast cancer that does not express the three receptors: estrogen receptor (ER), progesterone receptor (PR), and human epidermal growth factor receptor 2 (HER2)^1^. This renders it unresponsive primarily to traditional treatments such as chemotherapy, radiation, and biotherapy, thus making it difficult to treat. Approximately 15–20% of all breast cancer cases are TNBC, and the prognosis for this type is poor, with around a third of patients experiencing distant recurrences and death^1,2^. Consequently, discovering new biomarkers of TNBC can provide more tailored treatments, assess treatment responses, and predict prognostic statuses, thereby addressing an important area of unmet medical need^3–5^.

Gankyrin (also named PSMD10) is a proteasomal chaperone that plays a crucial role in the assembly and functioning of the proteasome^6,7^, as well as in the regulation of various oncogenic and inflammatory pathways through its protein–protein interactions^7–10^. Overexpression of gankyrin has been observed in several types of malignancies, such as hepatocellular carcinoma, cholangiocarcinoma, colorectal cancer, esophageal cancer, and breast cancer^10,11^. It has been established that gankyrin acts as a bridge between the proteasome and various tumor-associated substrates, such as p53, and can influence tumorigenesis by degrading tumor suppressor proteins and activating oncogenic signaling pathways^12–14^. Furthermore, gankyrin has been found to facilitate the binding of p53 to MDM2, a major E3 ubiquitin ligase, thus increasing ubiquitylation and degradation of p53. In the absence of p53, gankyrin has been observed to promote MDM2 autoubiquitylation and degradation^14,15^.

Ferroptosis is a form of programmed cell death distinct from apoptosis, necrosis, and autophagy in terms of its morphological, biochemical, and genetic characteristics. Recent studies have suggested that it may be a viable option for treating certain types of tumors, such as TNBC^16–18^, which have become resistant to traditional treatments^18–20^. The exact molecular mechanism of ferroptosis is still being investigated. Yet, it appears that the expression of proteins that inhibit ferroptosis, such as solute carrier family 7-member 11 (SLC7A11) and glutathione (GSH) peroxidase 4 (GPX4), is reduced in TNBC^21,22^. Additionally, p53 has been found to play a critical role in modulating ferroptosis in cancer cells^22^; SLC7A11 is a direct target gene suppressed by p53^23,24^. as it can directly restrict the SLC7A11 promoter region to block the transcription of SLC7A11, which encodes xCT, a sodium-independent cystine-glutamate antiporter. xCT accounts for the transportation of extracellular cystine into cells. Then cystine is reduced to cysteine used for glutathione (GSH) synthesis, which relies on GSH as its primary substrate, thus leading to cell ferroptosis^25^.

In this study, our results have revealed an unexplored link between gankyrin and p53, which plays a role in suppressing ferroptosis in TNBC cells. Our findings showed that gankyrin forms a complex with MDM2 and p53, that leads to the ubiquitination of p53 and an increase in SLC7A11 transcription. This, in turn, increases the production of GPX4 and enhances resistance to ferroptosis. The implications of our findings suggest that targeting gankyrin could be a viable therapeutic option for TNBC patients.

## Result

### Gankyrin is highly expressed in the TNBC tissues and cells, and is associated with the patient’s prognosis

**Figure 1:**
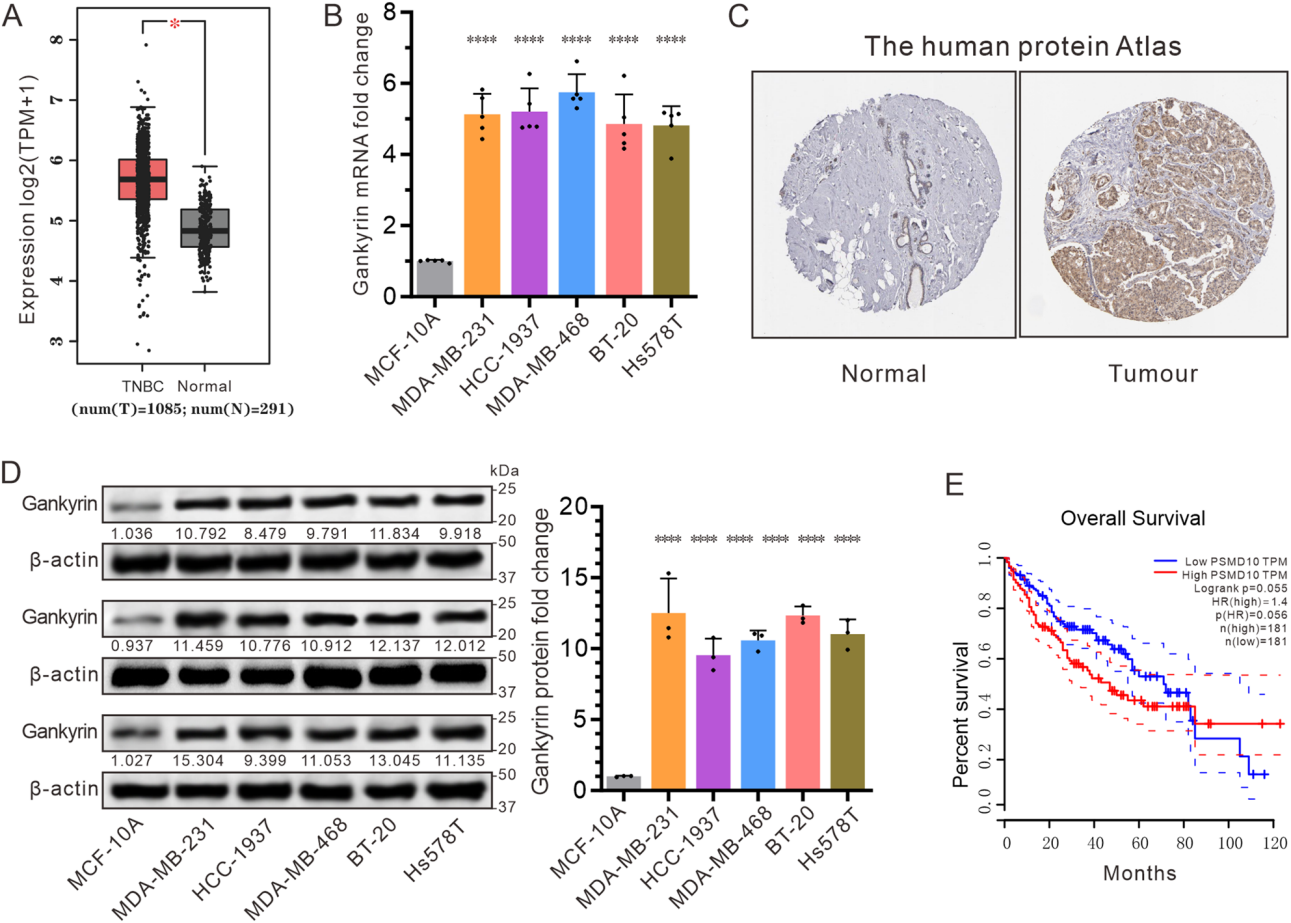
The expression of gankyrin is up-regulated in TNBC tissues and cells, and is negatively correlated with the patient’s prognosis. (**A**) Analysis of the GEPIA database indicates significantly higher levels of gankyrin in TNBC tumors compared to normal tissues. (**B**) The mRNA expression of gankyrin in various TNBC cell lines, including MCF-10A, MDA-MB-231, HCC-1937, MDA-MB-468, BT20, and Hs578T. (**C**) Protein mapping data highlights the differential expression of gankyrin in TNBC (tumor) and normal tissues. (**D**) The expression of gankyrin protein in a normal breast cell line and multiple TNBC cell lines as described in (**B**). (**E**) The GEPIA database demonstrates a contrast in overall survival rates between TNBC patients with high and low gankyrin expression. The data presented represents the mean ± standard deviation of three independent replicates and was analyzed using one-way univariate analysis of variance with multiple comparisons. Statistical significance was determined as *** < 0.001 and **** < 0.0001.

Studies have previously indicated that gankyrin plays a role in cell transformation and liver cancer formation^26^. To further explore its potential effect on TNBC, the Gene Expression Profiling Interactive Analysis (GEPIA) database was utilized to compare the expression of gankyrin in TNBC samples and multiple cancer cell lines to that of normal breast tissue or cells. Our results demonstrated a marked upregulation of gankyrin mRNA expression in TNBC (Fig. 1A,B). Additionally, immunohistochemical detection of paired samples from the same TNBC patient (HPA002920, patient ID: 2042) further demonstrated an increased gankyrin expression in cancerous tissue compared to normal breast tissue (Fig. 1C). Subsequently, the protein levels of gankyrin were examined in TNBC cell lines. Western blot results revealed an augmented expression of gankyrin in several TNBC cell lines, including MDA-MB-231, HCC-1937, MDA-MB-468, BT-20, Hs578T, compared to normal cell MCF-10A (Fig. 1D). Additionally, the prognostic significance of gankyrin expression was investigated in TNBC patients. Kaplan–Meier curves showed that increased gankyrin levels correlated with reduced overall survival; this notable disparity in patient survival times suggested a poor prognosis for breast cancer (Fig. 1E). Collectively, these results indicate that gankyrin is highly expressed in TNBC, and its heightened levels are linked to unfavorable outcomes in breast cancer patients.

### Small RNA interference against gankyrin promotes ferroptosis in TNBC cells

**Figure 2:**
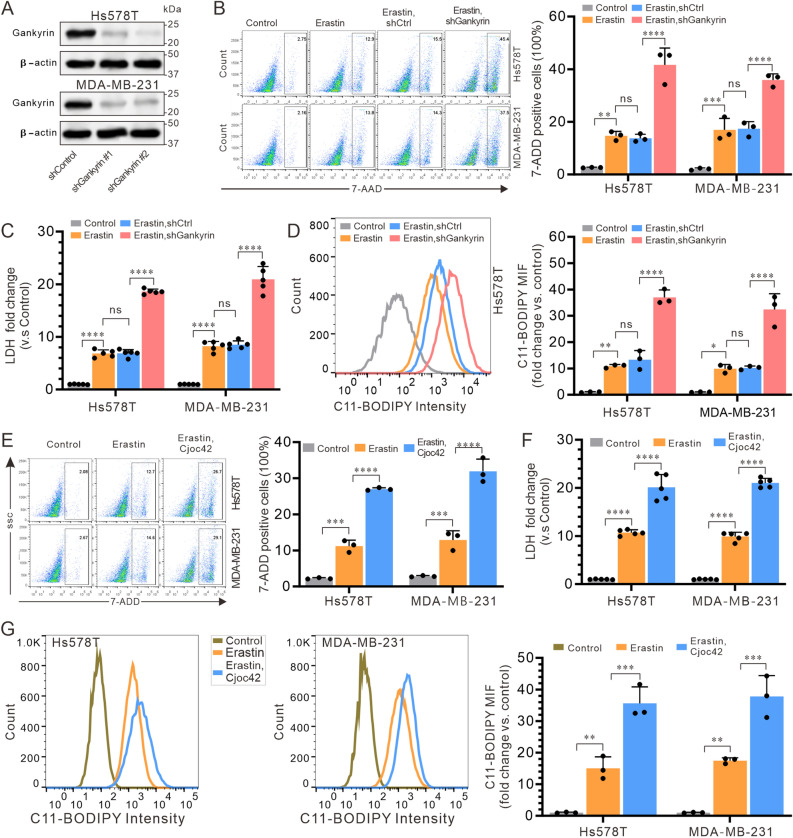
Overexpression of Gankyrin suppresses ferroptosis in TNBC cells. (**A**) Gankyrin protein expression in Hs578T and MDA-MB-231 cells transfected with shControl, shGankyrin #1, and shGankyrin #2 vectors, respectively. (**B**) Flow cytometry analysis of the proportion of 7-AAD-positive cells in Hs578T and MDA-MB-231 cells. (**C**) Fold changes in lactate dehydrogenase (LDH) release compared to the control. (**D**) Flow cytometry detection of lipid peroxides C11-BODIPY. (**E**–**G**) LDH release (**E**), proportion of 7-AAD-positive cells (**F**), and lipid peroxides C11-BODIPY (**G**) in Hs578T and MDA-MB-231 cells after gankyrin inhibition by cjoc42. Data represent the means ± standard deviations of three independent replicates and were analyzed using one-way univariate analysis of variance with multiple comparisons. Statistical significance was denoted as *** < 0.001 and **** < 0.0001.

We investigated whether gankyrin expression is associated with ferroptosis in TNBC cells by measuring levels of lactate dehydrogenase (LDH) release, 7-amino actinomycin D (7-AAD) stained cells, and lipid peroxide C11-BODIPY. To this end, we utilized small interfering RNA (siRNA) technology to suppress gankyrin expression in Hs578T and MB-MDA-231 cells, which was successful as evidenced by the decreased expression of gankyrin protein (Fig. 2A). The results showed that siRNA against gankyrin significantly increased cell death, as evidenced by a higher percentage of 7-AAD-positive cells (Fig. 2B) and increased LDH release (Fig. 2C) in the siRNA group than in the other groups. To further verify that this cell death was ferroptosis, we measured the level of C11-BODIPY by flow cytometry. The results indicated that, compared to the Control group, erastin treatment induced a significant increase of 7-AAD-positive cells (Fig. 2B), higher release of LDH (Fig. 2C), and a more pronounced generation of C11-BODIPY (Figs. 2D and S1A). Moreover, the gankyrin inhibitor Cjoc42 at a concentration of 5 μM was used to treat Hs578T and MB-MDA-231 cells. Consistently, Cjoc42 treatment resulted in a higher proportion of 7-AAD-positive cells (Fig. 2E), more increased LDH release (Fig. 2F), and a more pronounced generation of C11-BODIPY (Figs. 2G and S1B). Collectively, these findings demonstrate the critical role that gankyrin suppression plays in TNBC ferroptosis.

### Gankyrin negatively regulates the expression of p53 in TNBC cells

**Figure 3:**
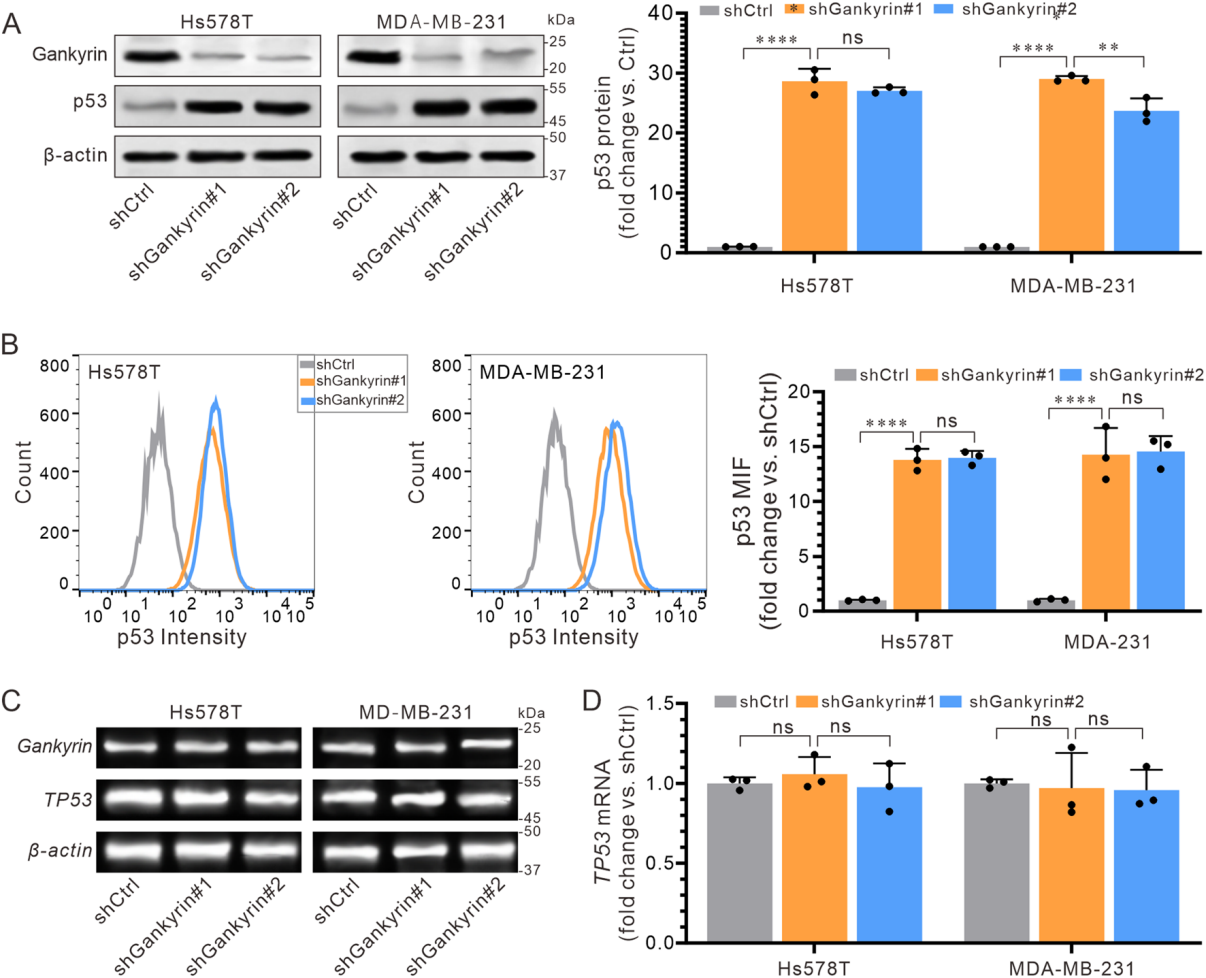
Effect of gankyrin on p53 protein expression in TNBC cells. (**A**) Levels of p53 protein expression in Hs578T and MDA-MB-231 cells after transfection with the designated siRNA interfering vectors. (**B**) Fluorescence intensity of p53 in Hs578T and MDA-MB-231 cells detected by Flow cytometry. (**C**) Expression of TP53 mRNA in Hs578T and MDA-MB-231 cells was detected by conventional RT-PCR. (**D**) Expression of TP53 mRNA in Hs578T and MDA-MB-231 cells was quantified by RT-qPCR. The results are presented as the mean ± standard deviation of three independent replicates and were analyzed using a one-way univariate analysis of variance with multiple comparisons. Statistical significance was determined as follows: ** < 0.01, *** < 0.001, **** < 0.0001, and ns indicating no statistical difference.

Evidence from studies on the transcriptional activity of the tumor suppressor gene p53 suggests that gankyrin has a negative regulatory effect on it^4,27^. To confirm this, we tested the effects of siRNA against gankyrin on the expression of p53 in Hs578T and MB-MDA-231 cells. Our results showed a marked increase in the protein expression of p53 (Fig. 3A), which was further confirmed by flow cytometry analysis (Figs. 3B and S2A). To investigate whether the suppression of p53 by gankyrin is related to transcription or translation, we conducted a normal RT-PCR to visualize and a RT-qPCR to quantitatively analyze the mRNA expression levels of p53, respectively. Our results showed that siRNA against gankyrin did not affect the expression of p53 mRNA, as detected by a normal RT-PCR (Fig. 3C) and RT-qPCR (Fig. 3D) analysis, implying that the inhibition of p53 by gankyrin is likely to be a post-transcriptional event.

### Suppression of p53 expression by gankyrin leads to inhibition of ferroptosis in TNBC cells

**Figure 4:**
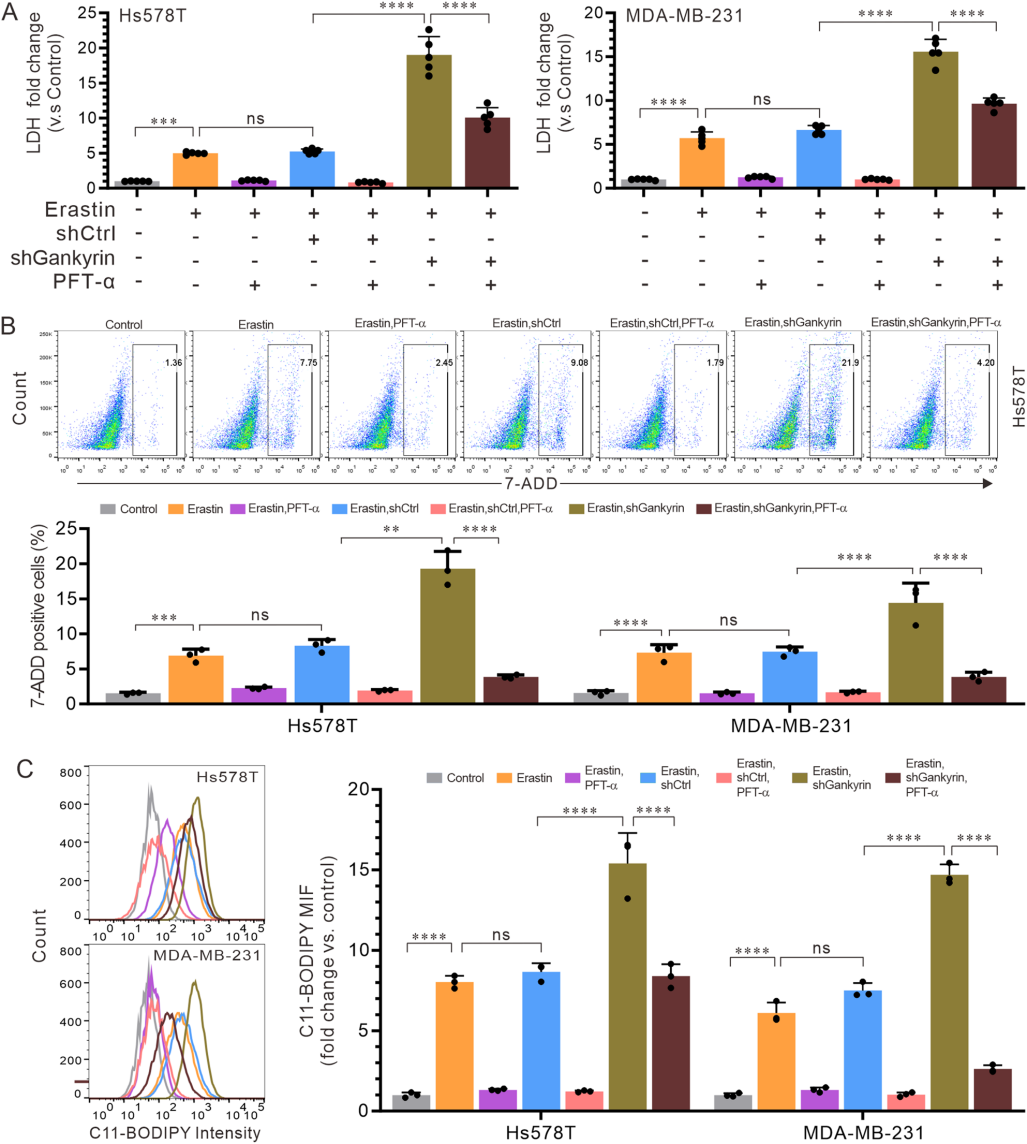
Involvement of p53 expression in the inhibition of ferroptosis. Hs578T and MDA-MB-231 cells treated with erastin were transfected with the shCtrl empty or shGankyrin expression vector for 24 h, followed by a 2-h incubation with or without the p53 inhibitor PFT-α (15 μM). (**A**) Fold changes in LDH release (vs. the shCtrl) were measured. (**B**) Proportion of 7-AAD positive cells in each group was analyzed by flow cytometry. (**C**) Lipid oxidates C11-BODIPY were analyzed by flow cytometry. The data presented represent the mean ± standard deviation of three independent replicates and were analyzed using one-way univariate analysis of variance with multiple comparisons. Statistical significance was determined as follows: ** < 0.01, *** < 0.001, **** < 0.0001, and “ns” indicating no statistical difference.

Recent evidence has implicated the p53 pathway in the regulation of ferroptosis^28^. It has been proved that increased expression of gankyrin can impede ferroptosis by degrading p53^29^. To investigate this, we conducted a co-treatment experiment involving siRNA targeting gankyrin, PFT-α (a known suppressor of p53), or erastin (a ferroptosis-inducing agent) on Hs578T and MB-MDA-231 cells. Our findings revealed that erastin treatment significantly increased LDH release (Fig. 4A) and the proportion of 7-AAD positive cells (Figs. 4B and S3A). Furthermore, the combination of erastin and siRNA against gankyrin led to an even greater LDH release (Fig. 4A) and an increased number of 7-AAD positive cells (Figs. 4B and S3A). However, these effects were partially attenuated by co-treatment with erastin, siRNA against gankyrin, and PFT-α (Fig. 4A,B). Our observations underscore that the attenuation of Gankyrin could amplify p53 expression, potentially facilitating ferroptosis in triple-negative breast cancer cells. Thus, we further clarified the relevance of Gankyrin to ferroptosis in these cells by inhibiting p53 expression. It was shown that the combination of erastin and siRNA targeting the Gankyrin protein resulted in more pronounced C11-BODIPY generation (Figs. 4C and S3B). However, the generation of C11-BODIPY decreased significantly with the incorporation of PFT-α. Taken together, these results suggest that inhibition of p53 expression by gankyrin can suppress ferroptosis in TNBC cells.

### Gankyrin overexpression accelerates MDM2-dependent ubiquitination-mediated degradation of p53

**Figure 5:**
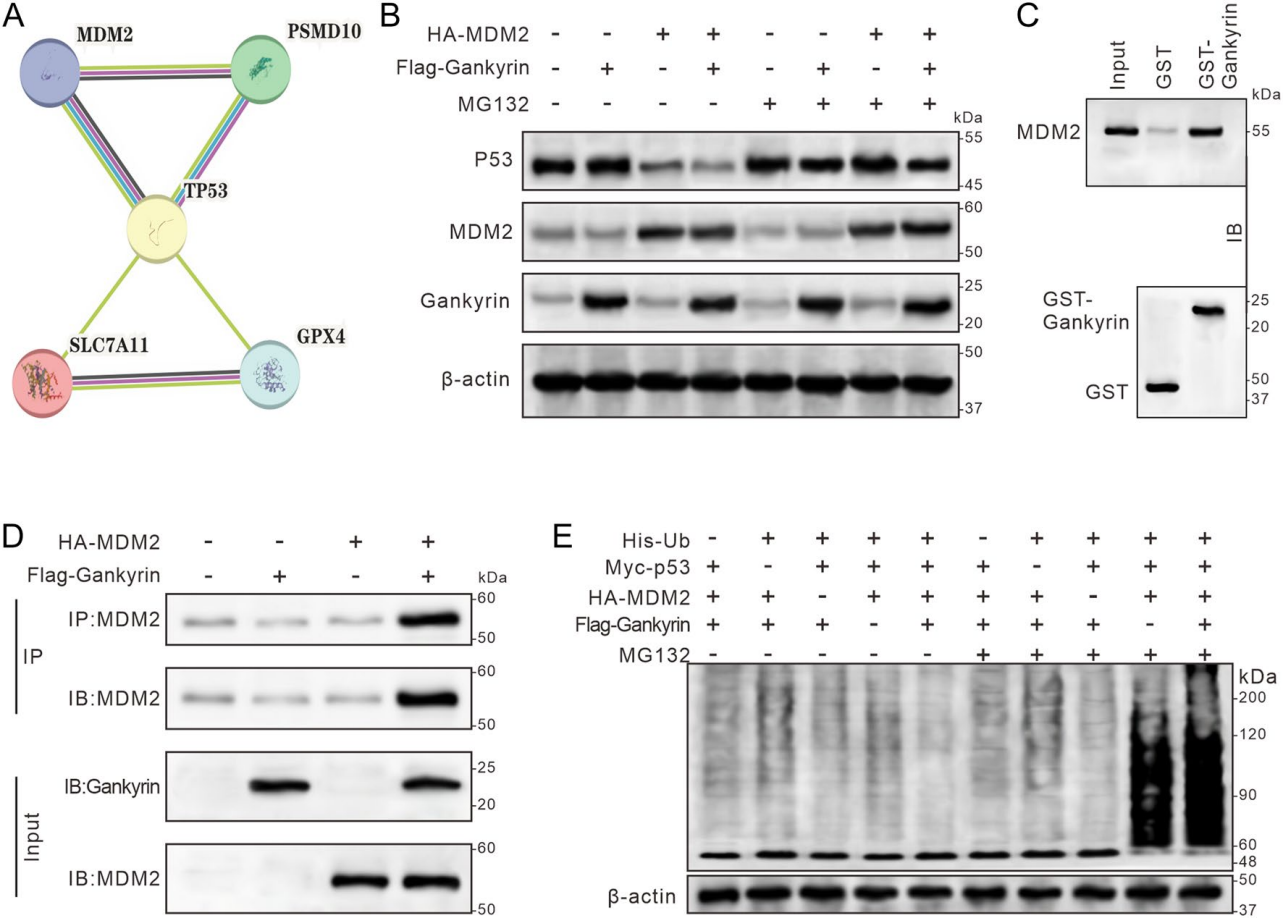
Gankyrin and MDM2 form a complex to promote ubiquitin-mediated degradation of p53. (**A**) STRING analysis reveals significant interactions among gankyrin, MDM2, p53, SLC7A11, and GPX4 proteins. (**B**) Expression levels of p53, MDM2, and gankyrin were assessed in HEK293T cells with a MDM2 knockout, transfected with MDM2 and gankyrin plasmids. (**C**) GST pull-down assay demonstrates the binding of GST-gankyrin to 35S-labeled MDM2 protein. (**D**) Immunoblotting using anti-gankyrin and anti-MDM2 antibodies confirms the interaction between gankyrin and MDM2 in the MDM2 knockout HEK293T cells. (**E**) Changes in p53 ubiquitination levels were measured by transfecting different combinations of Ub, p53, MDM2, and gankyrin plasmids in MDM2 and p53 double knockout MEF cells.

We conducted a pathway analysis using the online String database to gain insight into the mechanism behind the reduction of p53 protein levels by gankyrin. This analysis revealed intricate interactions between gankyrin, p53, MDM2, and ferroptosis-related molecules, such as GPX4 and SLC7A11 (Fig. 5A). Subsequent Western blot assays on mouse embryonic fibroblast cells (HEK293T) lacking the MDM2 gene showed that the expression of gankyrin or MDM2 accelerated the degradation of p53; however, the administration of the proteasome inhibitor MG132 abolished the p53 degradation by the expression of gankyrin or MDM2 (Fig. 5B). An immunoprecipitation assay further confirmed that MDM2 could only capture gankyrin when both proteins were co-expressed (Fig. 5C), which was further supported by the GST Pull-Down analysis (Fig. 5D). This indicated that MDM2 combined with gankyrin to form a complex. Furthermore, examination of ubiquitination ladders of p53 revealed that the co-expression of gankyrin and MDM2 induced p53 ubiquitination; this effect was not observed in the cells co-expressed with both MDM2 and gankyrin when the cells were not treated with MG132 (Fig. 5E). Taken together, these findings suggest that gankyrin overexpression accelerates MDM2-dependent ubiquitination-mediated degradation of p53.

### Gankyrin prevents ferroptosis by activating the p53/SLC7A11/GPX4 signaling axis in TNBC cells

**Figure 6:**
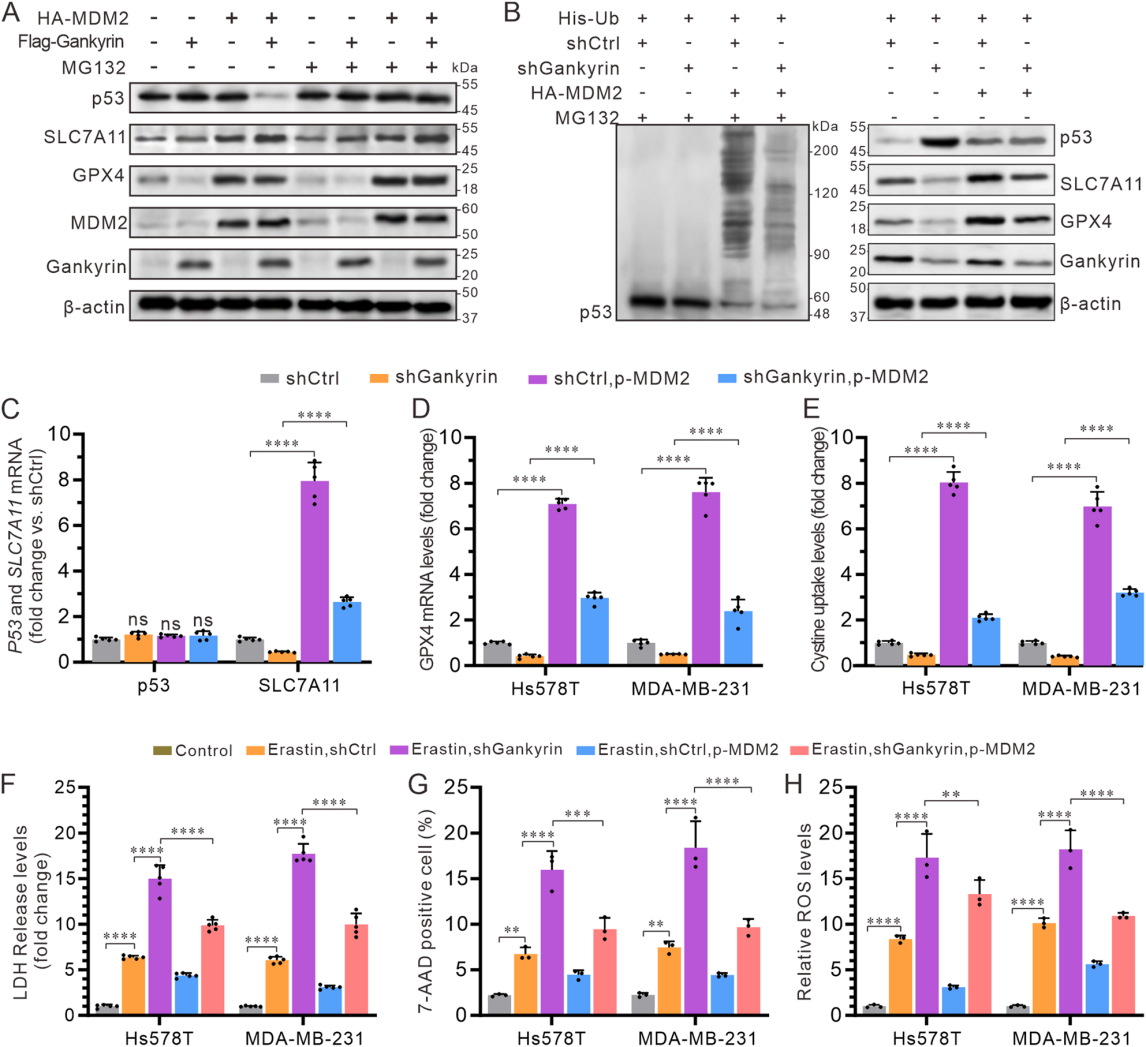
Role of PSMD10 in inhibiting ferroptosis in TNBC cells through the p53/SLC7A11/GPX4 pathway. (**A**) Protein expression of p53, SLC7A11, GPX4, MDM2, and gankyrin were examined in HEK293T cells expressing MDM2 with a HA-MDM2 vector. (**B**) Ubiquitination level of p53 (left panel) and the protein expression levels of p53, SLC7A11, GPX4, and gankyrin were assessed in Hs578T cells (right panel). (**C**) Hs578T and MDA-MB-231 cells were transfected with shGankyrin or shCtrl, along with a HA-MDM2 plasmid transfection, and the relative RNA expression levels of TP53 and SLC7A11 were measured. (**D**) Fold change in cystine uptake level compared to the shCtrl was determined. (**E**) Relative RNA expression level of GPX4 compared to the shCtrl was examined by RT-qPCR. (F) Fold change in LDH release compared to the control was measured. (**G** and **H**) Percentage of 7-AAD-positive dead cells (**G**) and the fold change in lipid peroxide C11-BODIPY compared to the control (**H**) were analyzed by flow cytometry. The data represent the mean ± SD from three independent experiments and were analyzed using two-way ANOVA followed by Tukey’s post-hoc multiple comparison analysis. Statistical significance is denoted as * < 0.05, ** < 0.01, *** < 0.001, **** < 0.0001. NS indicates no significance.

To gain a better understanding of the molecular pathways through which gankyrin affects ferroptosis and restricts cell proliferation in TNBC cells, we studied the connections between gankyrin, p53, MDM2, and the ferroptosis markers SLC7A11 and GPX4. In MDM2-deficient mouse embryonic fibroblast HEK293T, the presence of MDM2 alone resulted in a decrease in the expression of p53, accompanied by an increase in the expression of SLC7A11 and GPX4. However, when MDM2 and gankyrin were co-expressed, there was a further decrease in p53 expression, resulting in an even greater increase in SLC7A11 and GPX4 expression, and these effects were not affected by MG132 (Fig. 6A). To determine if the altered expression of SLC7A11 and GPX4 was linked to the ubiquitination of p53 in TNBC cells, we used siRNA against gankyrin in combination with the expression of MDM2 in Hs578T cells. As expected, the siRNA against gankyrin in combination with the expression of MDM2 led to a significant increase in p53 ubiquitination, which was accompanied by a marked increase in SLC7A11 and GPX4 expression (Fig. 6B).

To further explore the relationship between gankyrin, p53, MDM2, and ferroptosis, we used siRNA against gankyrin in combination with or without overexpression of MDM2 in Hs578T and MDA-231 cells. We then measured the mRNA expression of p53, SLC7A11, and GPX4, as well as cystine uptake. We found that, compared to the shCtrl and shGankyrin groups, overexpression of MDM2 did not affect the translation of p53 mRNA, but significantly increased the translation of SLC7A11 (Fig. 6C) and GPX4 (Fig. 6D) mRNA, as well as cystine uptake (Fig. 6E). However, co-treatment with siRNA against gankyrin and overexpression of MDM2 reversed the increase in mRNA expression of both SLC7A11 and GPX4, and the cystine uptake (Fig. 6C–E). Furthermore, erastin stimulation combined with siRNA against gankyrin induced the highest levels of ferroptosis in Hs578T and MDA-MB-231 cells, as evidenced by substantial LDH release (Fig. 6F), the highest proportion of 7-AAD-positive cells (Figs. 6G and S4A), and generation of lipid peroxide C11-BODIPY (Figs. 6H and S4B). These effects were counteracted by the overexpression of MDM2, and co-treatment with siRNA against gankyrin and overexpression of MDM2 partially rescued the LDH release (Fig. 6F), the proportion of 7-AAD-positive cells (Fig. 6G), and cystine uptake (Fig. 6H). Collectively, these results demonstrate that gankyrin can prevent ferroptosis by activating the p53/SLC7A11/GPX4 signaling axis in TNBC cells.

## Discussion

TNBC is a subtype of breast cancer that is associated with an increased risk of early distant recurrence and a poorer prognosis than other types of breast cancer^30^. Its heterogeneous nature and lack of defined molecular targets make it difficult to treat clinically^31–33^. Furthermore, TNBC displays both intra-tumoral and inter-tumoral heterogeneity, adding to the complexity of treatment strategies^33^. The lack of established therapeutic targets further complicates the management of TNBC^34,35^. Additionally, TNBC has a distinct metastatic pattern, often spreading to the brain and lungs^36^. Given the grave prognosis of TNBC, it is essential to develop systemic therapeutic approaches to improve patient survival rates. Therefore, the identification of possible molecular biomarkers is of paramount importance for TNBC diagnosis and the development of novel therapeutics.

Gankyrin, a protein related to oncogenesis, appears to be involved in the control of key signaling pathways and the destruction of regulatory molecules by the proteasome system^8^. Studies have indicated that its interaction with the E3 ubiquitin ligase MDM2 enhances the ubiquitination of p53 and its attachment to the S6b ATPase from the 19S regulatory particle of the proteasome, thus linking the ubiquitin–proteasome system to gene expression^13,29^. Moreover, overexpression of gankyrin has been linked to the metastasis of breast cancer, and its diminution in highly metastatic breast cancer cells has been seen to reduce cell migration and invasion^37^. Recent investigations have suggested that p53-mediated ferroptosis, a type of programmed cell death, might have tumor-suppressing activity^38,39^. However, the exact manner in which gankyrin regulates ferroptosis in TNBC cells is yet to be elucidated. In this research, it was determined that gankyrin is highly expressed in TNBC tissues and cells, and is inversely correlated with patient prognosis. Furthermore, small RNA interference targeting gankyrin was observed to induce ferroptosis in TNBC cells. It was also observed that gankyrin could form a complex with the E3 ubiquitin ligase MDM2, which then recruited p53, leading to its ubiquitination-mediated degradation. This decrease in the expression of ferroptosis-inhibiting molecules, such as SLC7A11 and GPX4, and reduced cystine uptake, resulted in the inhibition of ferroptosis in TNBC cells. This novel mechanism provides valuable insights into the potential for further research and treatment of TNBC.

Our research investigated the implications of gankyrin in tumorigenesis. Studies have demonstrated a correlation between an increase in gankyrin expression and advanced stages of cancer, as well as a poor prognosis, particularly in the case of TNBC^40,41^. Evidence has been presented to demonstrate its influence on multiple cancer-related pathways in various types of cancer. An elevation of gankyrin leads to the destruction of tumor suppressor proteins, resulting in an uncontrolled growth of cancer cells^27^. Our study revealed a significant increase in gankyrin expression in TNBC cells, indicating its critical role in the proliferation and growth of TNBC cells. Additionally, a high expression of gankyrin is significantly associated with a shorter survival period, which is in line with previous findings. These findings suggest that gankyrin may have a potential tumorigenic role in TNBC.

Gankyrin has been linked to multiple biochemical processes related to various diseases. Its increased expression has been correlated with decreased levels of p53, through an interaction with MDM2^13^. This has prompted the suggestion of inhibiting the interaction as a potential strategy to control the oncogenic effects of gankyrin overexpression^15^. Several E3 ubiquitin ligases have been identified as mediators of p53 degradation, including MDM2, COP1, Pirh2, and p300^12,42,43^. Our research demonstrated that the inhibition of gankyrin expression leads to an increase in p53 protein levels. This is in agreement with previous studies that showed that gankyrin interacts with MDM2, thus increasing its activity as a ubiquitin ligase towards p53. Additionally, we found that MDM2 binds directly to p53 to facilitate its ubiquitination-mediated degradation. However, the exact mechanism of gankyrin binding to MDM2 has yet to be elucidated, as the precise sites of gankyrin-MDM2 binding were not thoroughly investigated in this study.

Ferroptosis is a form of cell death that is the result of a disruption in the lipid oxidation metabolism and oxidative-regulated cell death^44,45^. This type of cell death is caused by a decrease in the cellular response to oxidative stress, leading to an increase of reactive oxygen species (ROS) levels^46^. It is thought that any factors that have an effect on the intracellular ROS levels can potentially lead to ferroptosis, and there is mounting evidence that it can inhibit cell growth in certain types of cancer^38^. Our research uncovered a significant correlation between gankyrin and ferroptosis in TNBC cells. We found that gankyrin expression induces ferroptosis, as evidenced by the increased ROS accumulation in erastin-treated Hs578T and MDA-MB-231 cells when gankyrin was knocked down. This was further supported by the results of small molecule inhibitors targeting gankyrin. At a molecular level, we explored the mechanisms connecting gankyrin and ferroptosis. We discovered that SLC7A11, a plasma membrane cystine/glutamate antiporter, plays a crucial role in this ferroptosis-induced cell death. Additionally, GPX4, a selenoprotein containing essential selenocysteine residues, functions downstream of SLC7A11^47^. It utilizes glutathione (GSH) as a cofactor to neutralize lipid peroxides, thereby preventing lipid-associated ROS accumulation and potentially leading to ferroptosis. Additionally, p53 suppresses cystine uptake, enhancing the cell’s susceptibility to ferroptosis^48,49^. We also observed that gankyrin expression increases p53 ubiquitination levels, which further amplifies ROS production. This eventually results in the inhibition of the SLC7A11/GPX4 axis and the promotion of ferroptosis in various tumor cells. In summary, our research has revealed a novel mechanism in which gankyrin increases the expression of SLC7A11 and GPX4 through p53 ubiquitination, thus hindering ferroptosis-induced cell death in TNBC and potentially facilitating cancer cell survival.

This study provides valuable insights into the role of gankyrin in triple-negative breast cancer and tumor ferroptosis. However, certain limitations must be taken into consideration. Firstly, our research only focused on the expression of gankyrin in triple-negative breast cancer, thus it is essential to determine if it is also expressed in other cell lines or tumors and to assess if it has a similar inhibitory effect on tumor ferroptosis. Previous studies have indicated that gankyrin is highly expressed in different tumors and is associated with poor prognosis in patients^10,11^, suggesting that it may play a role in suppressing ferroptosis in various tumor cells. Secondly, our attempts to use CRISPR-Cas9 technology for genetic manipulation and RNA interference technology for gene knockdown of gankyrin in several TNBC cell lines were unsuccessful, thus we cannot successfully establish tumor models for further investigation of the phenomenon in vivo. Lastly, our experimental design may be limited by our research experience, as the current results primarily reflect observable phenomena and the underlying molecular mechanism remains unclear. For instance, we lack evidence to support the hypothesis that gankyrin forms a complex with MDM2, leading to p53 degradation through ubiquitination. In conclusion, this study sheds light on the role of gankyrin in triple-negative breast cancer and tumor ferroptosis, yet further research is needed to expand our understanding of gankyrin in different cancer types, overcome technical challenges, and uncover the underlying molecular mechanisms.

In summary, this study highlights the significant role of gankyrin in the promotion of TNBC cells. Our results demonstrate that gankyrin suppresses p53-dependent ferroptosis in TNBCs and is essential for their proliferation. Notably, the overexpression of gankyrin in TNBC tissues and cells implies its capacity to affect cell fate. Further investigation revealed that the upregulation of gankyrin leads to ubiquitination-mediated degradation of p53, which then increases the expression of SLC7A11 and GPX4, resulting in the inhibition of ferroptosis. These findings demonstrate a complex regulatory mechanism by gankyrin, indicating that it may be a viable target for therapeutic treatment of TNBC.

## Materials and methods

### Cell culture and reagents cell culture and reagents

TNBC cell lines (MDA-MB-231, HCC-1937, MDA-MB-468, BT-20, and Hs578T) and normal breast epithelial cells (MCF10A) were obtained from the Cell Lines Service (Procell Co., Ltd., Wuhan, China) and authenticated by short tandem repeats (STR). MDM2 gene knockout HEK293T cells were purchased from Shanghai Binsui Biotechnology Co., Ltd., while 293 T cells used for lentiviral vector packaging were obtained from the American Type Culture Collection. All cells were cultured under standard conditions (37 °C and 5% CO_2_) in Dulbecco’s Modified Eagle’s Medium (DMEM, Gibco) supplemented with 10% fetal bovine serum (FBS). p53/MDM2 double-knockout mouse embryonic fibroblasts (p53 and MDM2 double-knockout mouse embryonic fibroblasts) were also cultured in DMEM supplemented with 10% FBS.

### Gankyrin gene expression and Kaplan–Meier plotter analysis in TNBC patients

The data and characteristic images for immunohistochemical analysis of gankyrin expression in normal breast tissue and TNBC tumor tissues were obtained from the Human Protein Atlas, a publicly available database (https://www.proteinatlas.org). Gene expression data and clinical data associated with overall survival in TNBC patients were retrieved from the Cancer Genome Atlas Program (TCGA) and can be accessed through the Gene Expression Profiling Interactive Analysis (GEPIA) website (http://gepia.cancer-pku.cn/detail.php?gene=foxm1). To obtain data specific to TNBC, one can input the gene name “Gankyrin” and select the tumor type TNBC within the website following the website’s instructions.

### RT-PCR

Total RNA was extracted from the cells using TRIzol (TRlzol; 15,596-018; Invitrogen) and treated with DNase I to eliminate DNA contamination. Subsequently, single-stranded cDNA was synthesized from the RNA using reverse transcriptase M-MLV (D2640A, TaKaRa, Dalian, China). Primer sequences for the quantification of gankyrin, p53, SLC7A11, GPX4, and β-actin mRNA expression were provided in Table S1, which were synthesized by TaKaRa. Subsequently, all RT-qPCR analysis was conducted with SYBR Green Master Mix to measure the mRNA expression levels of gankyrin, p53, and GPX4. The PCR reaction conditions were based on a published report^50^. All RT-qPCR analysis was performed using rTaq (TaKaRa) in a DNA thermal cycler (Maxygen). Additionally, another set of primers (Table S2) was used to perform a normal RT-PCR to detect the expression of mRNA of gankyrin, p53, and β-actin, as previously reported^51^, using a gradient thermal cycler (Tgradient 96, Biometra, Germany). The images were then captured by an automatic digital gel image analysis system (Tanon-4100, Tanon, Shanghai, China).

### Design and synthesis of the gankyrin shRNA vectors

The Invitrogen online RNAi Designer was utilized to design two optimal shRNAs and a control scrambled shRNA against the mRNA sequence of human gankyrin (GenBank; NM_002814.3). The two complementary single-stranded DNA oligonucleotides of the three shRNAs were chemically synthesized by TaKaRa and annealed to form double-stranded oligonucleotides (Table S3). The shRNA vectors shGankyrin and the control scrambled shRNA vector shControl were then transformed into competent DH5α cells (catalog no. 9057) to obtain enough shRNA vectors for subsequent experiments as previously reported^52^.

### Flow cytometry

Flow cytometry was utilized to quantify p53-expressing cells, 7-aminoactinomycin D (7-AAD) positive death cells, and lipid peroxidation cells, as previously detailed^53,54^. In brief, to measure the expression of p53 in Hs578T and MDA-MB-231 cells, two million cells of Hs578T and MDA-MB-231 were fixed with 0.25% cold paraformaldehyde for 1 h at 4 °C, followed by lysis with 0.1% Triton X-100 (Thermo Scientific). The cells were then incubated with the p53 antibody DO-7 (GA61661-2, Dako, KBH, DNK) at room temperature for an hour, and analyzed using a flow cytometer. To measure 7-AAD positive death cells, cells were incubated with 20 μM of the ferroptosis inducer erastin for 24 h, and then resuspended in PBS containing 1 μg/ml of 7-AAD for 10 min, which were then applied to calculate the percentage of 7-AAD positive cells by flow cytometry. Additionally, BODIPY-C11 lipid peroxide was also detected by flow cytometry as previously reported. In brief, Hs578T and MDA-MB-231 cells were seeded in triplicates in 12-well plates for 24 h, followed by treatment with test compounds for the specified duration. Subsequently, cells were incubated with fresh medium containing 2 μm BODIPY 581/591C11 dye (Invitrogen) at 37 °C for 20 min. After trypsinization, cells were washed with PBS by centrifugation, and the fluorescence intensity of cells with BODIPY 581/591C11 staining was measured by flow cytometry. The fold change of the mean fluorescence intensity (MFI) over the control group (Ctrl) was then calculated for each sample. All the flow cytometry analyses were performed by the CyFlow Cube 6 system (Sysmex, Kobe, Japan) and then analyzed and images captured by FlowJo software (BD Biosciences).

### Western blot analysis

Cells were initially washed with cold phosphate-buffered saline (PBS) from Thermo Fisher Scientific (US). Total protein was extracted with a combination of RIPA buffer (Sigma Aldrich, US) and a protease inhibitor cocktail (Roche, Shanghai, CN) per the manufacturer’s instructions. 25–30 mg of protein were loaded onto a 4–12% SDS-PAGE gel (BeyoGel, CN), electrophoresed at 120 V for 1 h, and then transferred onto a PVDF membrane at 250 mA for 2 h. Subsequently, we pre-cut the PVDF membrane into sections according to the molecular weight of the target proteins, thereby decreasing the amount of incubation solution used during the antibody incubation step, leading to a cost saving as the use of costly antibodies is reduced. Following this, a 30-min blocking with 5% non-fat milk was performed. The membrane was then incubated overnight at 4 °C with primary antibodies, including a rabbit polyclonal anti-gankyrin antibody (Santa Cruz Biotechnology)^55^, a mouse monoclonal anti-p53 antibody (DO-7, GA61661-2, Dako, KBH, DNK)^53^, a mouse monoclonal anti-MDM2 antibody (SMP14 Santa Cruz)^12^, a rabbit monoclonal Anti-SLC7A11 (ab37185 Abcam)^56^, and a rabbit monoclonal Anti-Glutathione Peroxidase 4 (ab125066, Abcam)^56^. Finally, goat anti-rabbit IgG or anti-mouse IgG (Zymed, San Francisco, CA) was utilized as secondary antibodies^55^. Western blot images were captured using an ECL imaging system from Thermo Fisher Scientific (US)^50^.

### Lactate dehydrogenase release experiment

Per the manufacturer’s instructions, the LDH release assay kit (Beyotime, C0016) assessed lactate dehydrogenase (LDH) levels from Hs578T and MDA-MB-231 cells. In brief, 100 μL/well of cells with a concentration of 10^5^ cells/mL were added to a 96-well plate with 20 μL of 0.4 mol/L lactic acid solution, 20 μL of 4 mmol/L 2-p-iodophenyl-3-p-chloronitrobenzenetetrazole, and 20 μL of reaction solution. Subsequently, the samples were incubated at room temperature for 30 min, and the optical density (OD) value was measured using an ELISA reader (ELX808IU, Bio-Tek) with a detection wavelength of 492 nm and a reference wavelength of 650 nm. The fold change of each sample (including the control group sample) was computed by dividing the value of each sample with the mean value of the control group, as previously reported.

### Co-immunoprecipitation assay

Immunoprecipitation was performed on a cell lysate of MDM2 KO HEK293T cells that had been co-transfected with an HA-tagged MDM2 plasmid and a Flag-tagged Gankyrin plasmid. To begin, these HEK293T cells were washed twice with chilled PBS and then lysed with a co-IP buffer. The lysate was then incubated overnight at 4 °C with Agarose Conjugated Anti-HA Mouse Monoclonal Antibody (ABT2043, Abbkine, CA) and Agarose Conjugated Anti-Flag Mouse Monoclonal Antibody (ABT2013, Abbkine, CA). This was followed by a 2-h incubation with Protein G Sepharose (Santa Cruz Biotechnology, sc-2002, CA). Afterwards, protein A/G beads were added and allowed to co-incubate for two s at 4 °C. The immunocomplexes containing the target proteins were then detected by WB, using specific antibodies against the non-target protein of interest. To further investigate the ubiquitination of p53, the cells were treated with MG132 and other reagents before the immune complexes were washed three times with PBS and subjected to WB analysis using Flag (AF519, Beyotime, Shanghai, China) and HA (AH158, Beyotime, Shanghai, China) antibodies^12^.

### GST-pull down assay

The GST-Gankyrin fusion protein was cloned into the expression vector pGEX-4T-1 (44,982,352,628, SHBCC, China) and isolated from a BL21 DE3 bacterial culture obtained from Boster Biotechnology (Wuhan, China) and immobilized on Glutathione-Sepharose (C4-0531-01-03, Seplife, Xian, China). The extraction process followed the instructions provided by the manufacturer. To minimize non-specific interference, the fusion proteins underwent treatment with DNase and RNase A. MDM2 protein was obtained from a rabbit reticulocyte lysate system (L4600, Promega, WI) and 35S-labeled methionine (JW-S53290, GIVEI, Shanghai, China) was added to the translation process. The in vitro translated 35S-labeled MDM2 protein was then added to the immobilized GST-Gankyrin and GST and incubated at 4 °C for an hour. The bound proteins were eluted using an elution buffer containing 5 mM reduced glutathione, 10 mM Tris–HCl (pH 8.0), and 150 mM NaCl. For the preparation of cellular lysates, the cells were washed twice with icy PBS, and included a protease inhibitor cocktail from Sigma-Aldrich. Following lysis, the lysates were clarified through centrifugation and supplemented with 0.5% bovine serum albumin as a non-specific competitor. It is noteworthy that this protocol closely resembles the GST pull-down assay that had previously been described^57^.

### Plasmids and transfection for the detection of ubiquitination

Human Gankyrin, p53, MDM2, and Ub full-length cDNAs were synthesized by Invitrogen and cloned into plasmids pCMV-T7-MCS-3 × FLAG-WPRE-Neo (Addgene), pCMV-MCS-3 × Myc-Neo (Addgene), pCMV-MCS-3 × HA-Neo (Solarbio), and pCDNA3.1-MCS-6 × His (Invitrogen), respectively. This resulted in the production of Flag-Gankyrin, Myc-p53, HA-MDM2, and His-Ub plasmids. These plasmids were then used to transfect mouse embryonic fibroblasts with a double-knockout of p53 and MDM2 either alone or in combination for related experiments as previously reported^58,59^. In brief, mouse embryonic fibroblasts with a double-knockout of p53 and MDM2 in the logarithmic growth phase were first enzymatically dissociated using trypsin and then quantified. These cells were then plated into a six-well culture dish and when the cellular confluence reached 70%, they were transfected with the four plasmids individually or in combination by using Lipofectamine 2000 (Invitrogen) as per the manufacturer’s instructions. After 48 h of transfection, the cells were exposed to 25 μM MG132 (M8699, Sigma-Aldrich, MO) for 6 h and lysed with RIPA buffer. Subsequently, an immunoblotting assay using a p53-specific antibody (DO-7) was conducted according to the protocol for Western blot analysis described above to measure the p53 protein level in each sample. Lastly, the ubiquitination level of the p53 protein was observed and the images were captured with an ECL imaging system from Thermo Fisher Scientific (US).

### Cystine uptake assay

The Cystine Uptake Assay Kit (UP05, DOJINDO, Kumamoto Prefecture, Japan) was employed to measure the cystine uptake level, in accordance with the manufacturer’s instructions. In brief, cells were plated onto a black 96-well plate and incubated overnight. Subsequently, the culture medium was removed and the cells were washed three times with a serum-free DMEM medium that did not contain cysteine. Each well was then treated with 200 μL of DMEM medium containing either 0 μMol/L Erastin (Sample 1, Blank) or 100 μMol/L Erastin (Sample 2). The plate was placed in a 5% CO_2_ incubator for 5 min. After the incubation period, the supernatant was removed from each well and 200 μL of CA uptake solution at 37 °C was added. The plate was then placed back into the 5% CO_2_ incubator and incubated for an additional 30 min. Following this, the supernatant was again removed and the cells were washed three times with chilled PBS. To extract the Cytosine Analog absorbed by the cells, each well was treated with 50 μL of methanol and mixed thoroughly. The wells were then covered with a sealing film and incubated in a 5% CO_2_ incubator for 30 min. Fluorescence detection was carried out using a fluorescence enzyme-linked immunosorbent ELISA reader (ELX808IU, Bio-Tek) with an excitation wavelength of 490 nm and an emission wavelength of 535 nm. The fluorescence intensity generated by the Cytosine Analog uptake by the cells was calculated by subtracting the detection value of the blank sample from the detection value of the sample, following a previously described method^60^.

### Statistical analysis

Data analysis was performed using GraphPad Prism software version 9.0.0 for Windows (GraphPad Software, San Diego, California, USA). Data were presented as the mean ± standard deviation (SD). Group differences were analyzed using one-way or two-way analysis of variances (ANOVA), followed by Tukey’s post hoc multiple comparison tests. An adjusted *P* value of less than 0.05 was considered statistically significant.

### Supplementary Information

Supplementary Information 1.

## Data Availability

Data supporting the findings of this study are available in this paper, Supplementary material, or are available from the corresponding author upon request.

## Abbreviations

7-AAD: 7-Aminoactinomycin D
Co-IP: Co-immunoprecipitation
ECL: Enhanced chemiluminescence
ELISA: Enzyme-linked immunosorbent assay
ER: Estrogen receptor
FBS: Fetal bovine serum
FCM: Flow cytometry
FITC: Fluorescein isothiocyanate
GEPIA: The gene expression profiling interactive analysis
GPX4: Glutathione peroxidase 4
GSH: Glutathione
GST: Glutathione-S-transferase
Her-2: Human epidermalgrowth factor receptor-2
IgG: Immunoglobulin G
LDH: Lactate dehydrogenase
MDM2: Mouse double minute 2
MFI: Mean fluorescence intensity
M-MLV: Moloney Murine Leukemia Virus
MOI: Multiplicity of infection
PBS: Phosphate-buffered saline
PR: Progesterone receptor
ROS: Reactive oxygen species
RT-qPCR: Reverse-transcription quantitative real-time polymerase chain reaction
SDS-PAGE: Sodium dodecyl sulfate polyacrylamide gel electrophoresis
SLC7A11: Solute carrier family 7 member 11
STR: Short tandem repeat
TNBC: Triple-negative breast cancer
TRIzol: A chemical solution used in the extraction of DNA
Ub: Ubiquitination
WB: Western blotting
xCT: System xc-cystine/glutamate antiporter
