# Supplementary material for: Gankyrin inhibits ferroptosis through the p53/SLC7A11/GPX4 axis in triple-negative breast cancer cells
Source: Sci Rep. 2023 Dec 8;13:21916. doi: 10.1038/s41598-023-49136-8 (PMC10713534; doi:10.1038/s41598-023-49136-8)
Supplement: Supplementary file 1 — Supplementary Information 1. [file 41598_2023_49136_MOESM1_ESM.docx]

**Gankyrin inhibits ferroptosis through the p53/SLC7A11/GPX4 axis in Triple-negative breast cancer cells**

Ming Lei^1,2,*^，Yun-long Zhang^2,*^, Feng-Ying Huang^2,*^, Heng-Yu Chen^1^, Ming-Hui Chen^2^, Ri-Hong Wu^2^, Shu-Zhen Dai^2^, Gui-Sheng He^1,#^, Guang-Hong Tan^2,#^, Wu-Ping Zheng^1,#^

^1^Department of Breast and Thyroid Surgery, The Second Affiliated Hospital, Hainan Medical University, Haikou 570311, China.

^2^Key Laboratory of Tropical Translational Medicine of Ministry of Education & School of Tropical Medicine, Hainan Medical University, Haikou 571199, China.

* These authors contributed equally to the work

^#^Corresponding Authors:

Wu-Ping Zheng: [hnzwp2000@163.com](mailto:hnzwp2000@163.com)

Gui-Sheng He: [512688772@qq.com](mailto:512688772@qq.com)

Guang-Hong Tan: [tanhoho@163.com](mailto:tanhoho@163.com)

**Supplementary Figures and legends**


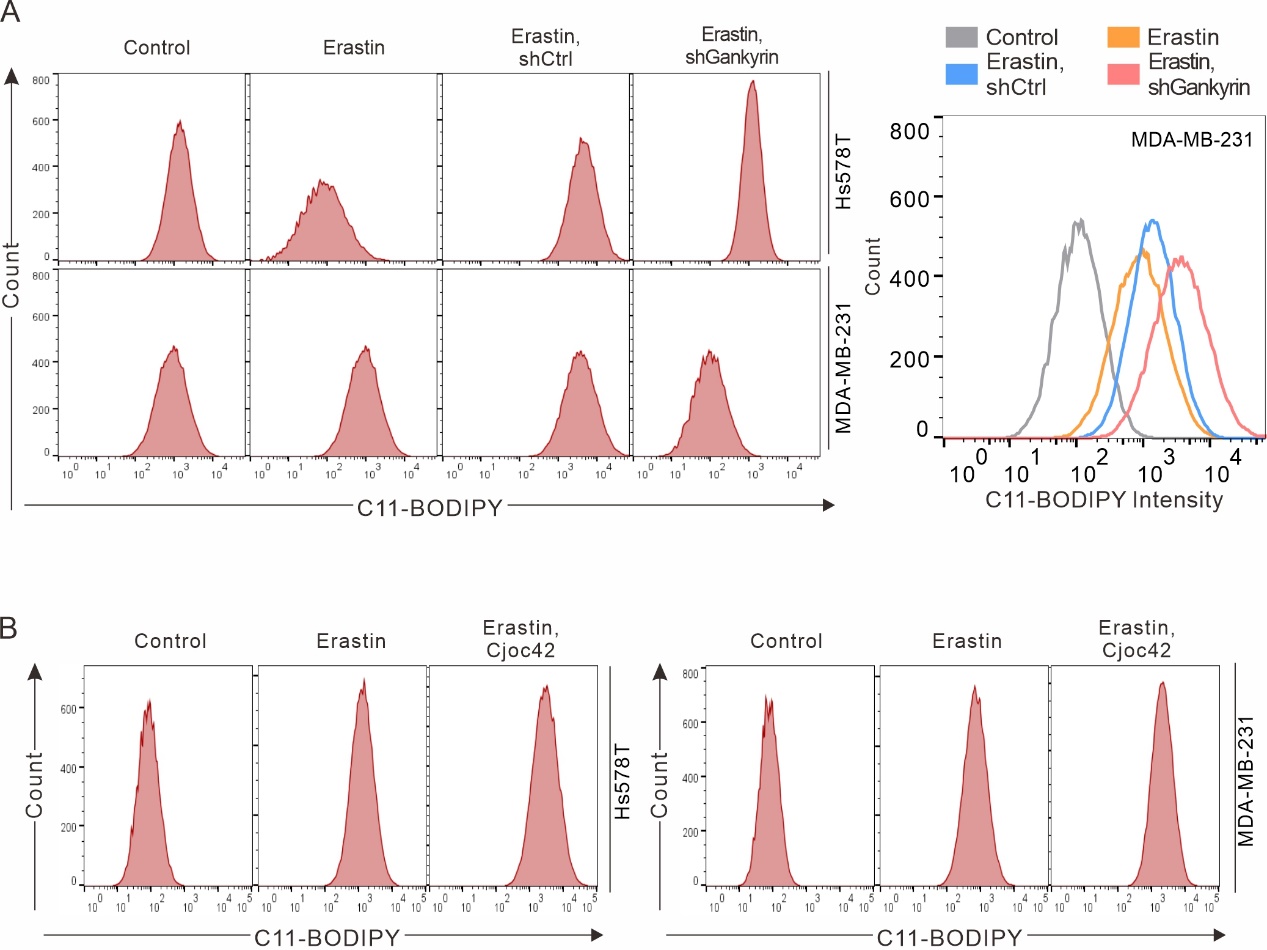


**Figure S1. (A)** Representative images of the flow cytometry results from three repeated experiments, related to Figure 2D. **(B)** Representative images of the flow cytometry results from three repeated experiments, related to Figure 2G.

**
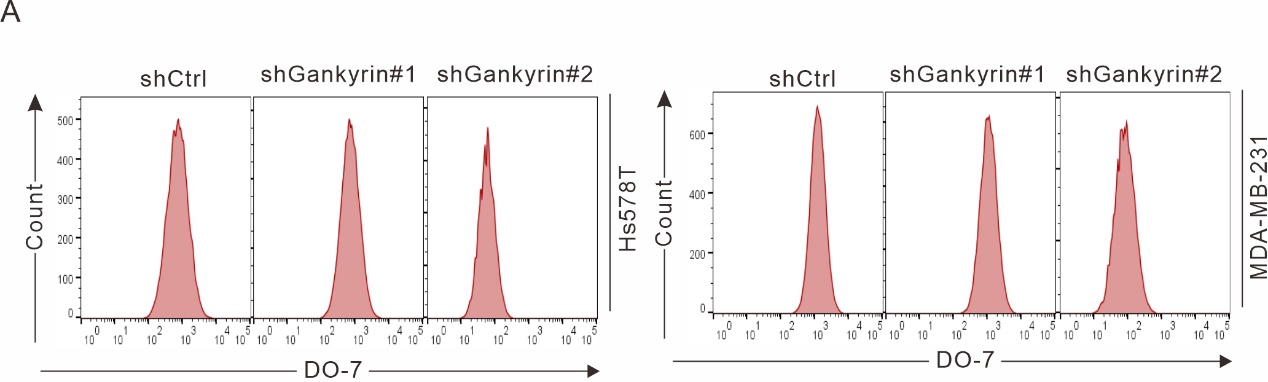
**

**Figure S2. (A)** Representative images of the flow cytometry results from three repeated experiments, related to Figure 3B.

**
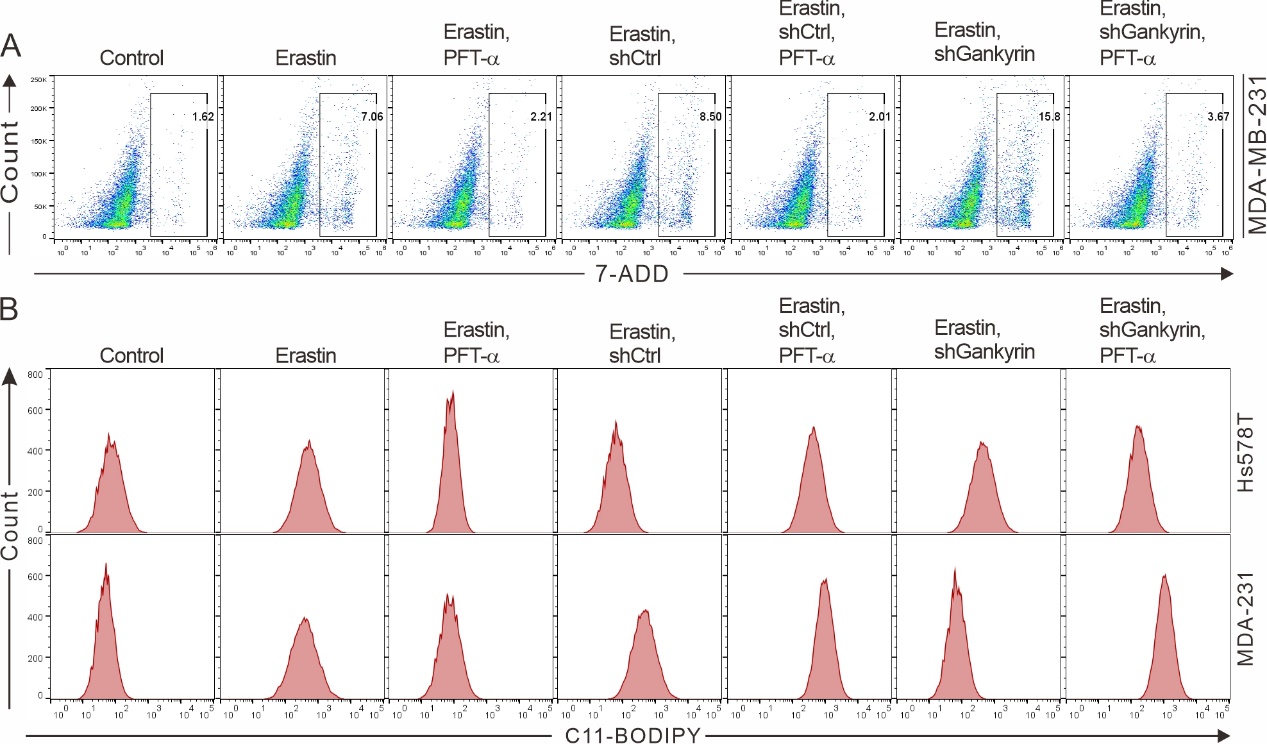
**

**Figure S3. (A)** Representative images of the flow cytometry results from three repeated experiments, related to Figure 4B. **(B)** Representative images of the flow cytometry results from three repeated experiments, related to Figure 4C.

**
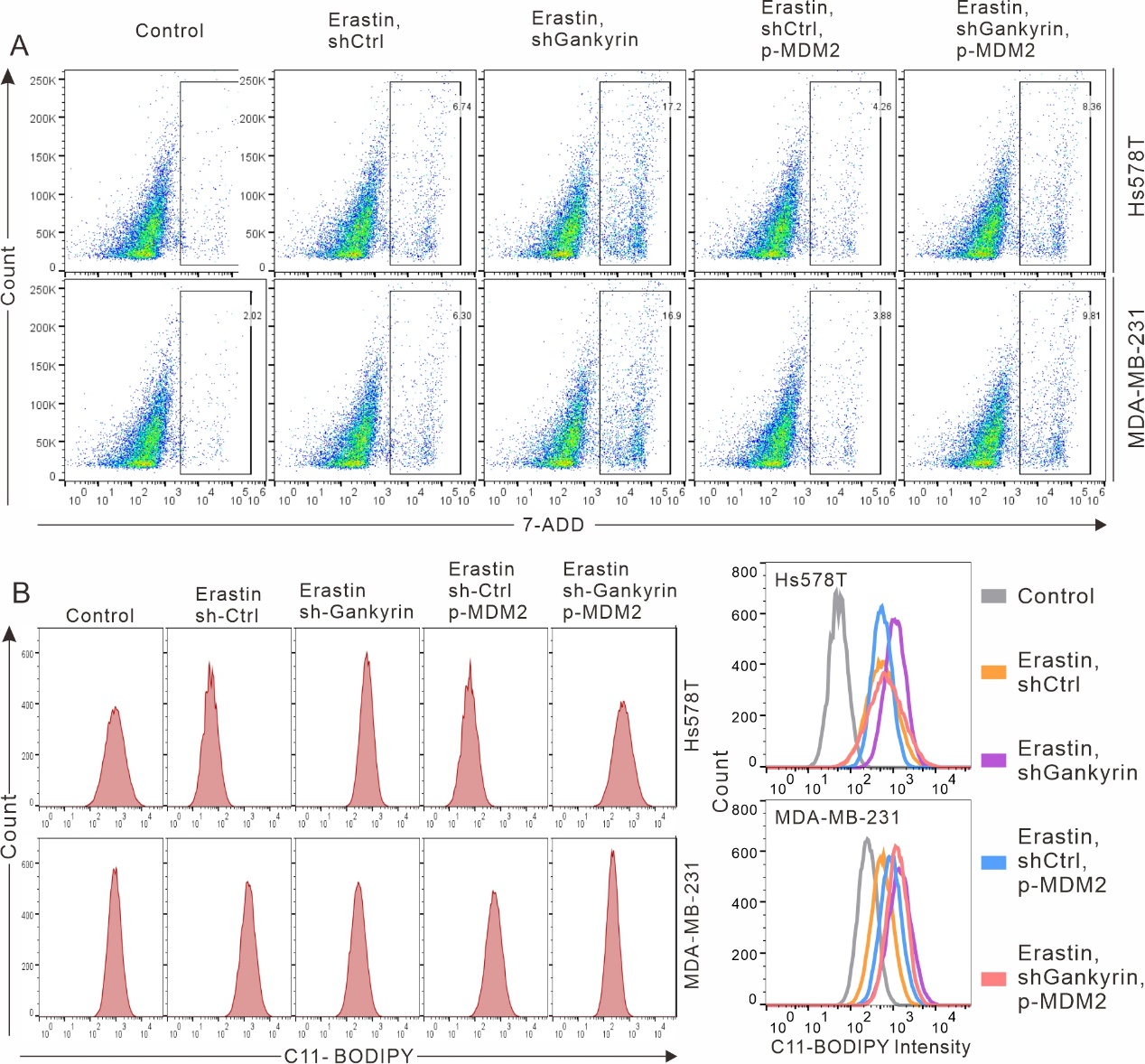
**

**Figure S4. (A)** Representative images of the flow cytometry results from three repeated experiments, related to Figure 6G. **(B)** Representative images of the flow cytometry results from three repeated experiments, related to Figure 6H.

**Table S1:** Primers Used for RT-qPCR Reaction

| **Gene** | **Forward (5’-3’)** | **Reverse (5’-3’)** |
| --- | --- | --- |
| Gankyrin | CTGGCCGGGATGAGATTGTAAAAG | CGGTGCATTGCTGTAGCCTCATAA |
| P53 | CACGAGCTGCCCCCAGG | TCAGTCGACGTCTGAGT |
| SLC7A11 | GCTGTGATATCCCTGGCATT | GGCGTCTTTAAAGTTCTGCG |
| GPX4 | AGAGATCAAAGAGTTCGCCGC | TCTTCATCCACTTCCACAGCG |
| β-actin | CCCAAGGCCAACCGCGAGAAGATG | GTCCCGGCCAGCCAGGTCCAGA |

**Table S2:** Primers Used for RT-PCR Reaction

| **Gene** | **Forward (5’-3’)** | **Reverse (5’-3’)** |
| --- | --- | --- |
| Gankyrin | AGCAGCCAAGGGTAACTTGA | TACTTGCTCCTTGGGACACC |
| P53 | CTGAGGTTGGCTCTGACTGTACCACCATCC | CTCATTCAGCTCTCGGAACATCTCGAAGCG |
| β-actin | TACTGAAGTGTGACGTGGACATC | CAGGAGGAGCAATGATCTTGATCT |

**Table S3：**Sequences of 3 designed shRNA-Gankyrin

| **Gene** | **Sequences** |
| --- | --- |
| shRNA1-f | 5’-GATCCGCCGATAAATCCCTGGCTACTTCCTGTCAGATAGCCAGGGATTTATCGGCTTTTTG-3’ |
| shRNA1-r | 5’-AATTCAAAAAGCCGATAAATCCCTGGCTATCTGACAGGAAGTAGCCAGGGATTTATCGGCC-3’ |
| shRNA2-f | 5’-GATCCGGCTGTACTCCCTTACATTCTTCCTGTCAGAAATGTAAGGGAGTACAGCCTTTTTG-3’ |
| shRNA2-r | 5’-AATTCAAAAAGGCTGTACTCCCTTACATTTCTGACAGGAAGAATGTAAGGGAGTACAGCCC-3’ |
| NC-f | 5’-GATCCGAAGCCAGATCCAGCTTCCCTTCCTGTCAGAGGAAGCTGGATCTGGCTTCTTTTTG-3’ |
| NC-r | 5-AATTCAAAAAGAAGCCAGATCCAGCTTCCTCTGACAGGAAGGGAAGCTGGATCTGGCTTCC-3’ |

**Abbreviations:**f, forward; r, reverse; shRNA, short hairpin RNA.
